# Autoinducer-2 promotes *Pseudomonas aeruginosa* PAO1 acute lung infection *via* the IL-17A pathway

**DOI:** 10.3389/fmicb.2022.948646

**Published:** 2022-08-10

**Authors:** Hongdong Li, Xingyuan Li, Qing Ai, Liping Tan

**Affiliations:** ^1^Department of Emergency, Children's Hospital of Chongqing Medical University, Chongqing, China; Ministry of Education Key Laboratory of Child Development and Disorders, Chongqing, China; National Clinical Research Center for Child Health and Disorders, Chongqing, China; China International Science and Technology Cooperation Base of Child Development and Critical Disorders, Chongqing, China; Chongqing Key Laboratory of Child Infection and Immunity, Chongqing, China; ^2^Department of Pharmacy, Chongqing Red Cross Hospital, Chongqing, China

**Keywords:** *Pseudomonas aeruginosa*, autoinducer-2, Th17 cell, interleukin-17A, infection

## Abstract

*Pseudomonas aeruginosa* is an opportunistic pathogenic bacterium that causes various acute and chronic lung infections in immunocompromised patients. We previously found that a quorum sensing (QS) signal, namely, autoinducer-2 (AI-2), facilitates the pathogenicity of the wild-type (WT) *P. aeruginosa* PAO1 strain *in vitro* and *in vivo*. However, the immunological mechanism that leads to pulmonary injury remains to be elucidated. In this study, we aimed to investigate the effects of AI-2 on interleukin-17A (IL-17A) production during acute *P. aeruginosa* PAO1 lung infection using a mouse model, with an emphasis on the underlying immunological mechanism. Compared to infection with *P. aeruginosa* PAO1 alone, infection with *P. aeruginosa* PAO1 combined with AI-2 treatment resulted in significantly increased levels of IL-17A, numbers of Th17 cells and levels of STAT3 in the lung tissues of WT mice (*P* < 0.05), as well as more serious lung damage. In contrast, the concentrations of the proinflammatory cytokines IL-1α, IL-1β, and IL-6 and the chemokine keratinocyte-derived chemokine (KC) were significantly reduced during *P. aeruginosa* lung infection in IL-17A^−/−^ mice compared with WT mice (*P* < 0.05), and no effects were observed after AI-2 treatment (*P* > 0.05). Furthermore, the level of IL-17A in the lungs of WT mice was significantly reduced following infection with a *P. aeruginosa* strain harboring mutations in the QS genes *lasR* and *rhlR* compared with the level of IL-17A following infection with *P. aeruginosa* PAO1. Our data suggest that AI-2 promotes *P. aeruginosa* PAO1 acute lung infection *via* the IL-17A pathway by interfering with the QS systems of *P. aeruginosa*. IL-17A may be a therapeutic target for the treatment of acute *P. aeruginosa* lung infections in the clinic.

## Introduction

*Pseudomonas aeruginosa*, a ubiquitous gram-negative bacterium, is commonly found in the environment. In healthy individuals, *P. aeruginosa* can be cleared by host defenses. However, in immunocompromised patients, *P. aeruginosa* can be a formidable opportunistic pathogen that leads to various acute and chronic infections, such as acute or chronic pneumonia, bloodstream infections and other recalcitrant multidrug-resistant infections (Curran et al., 2018). We previously observed high diversity in the polymicrobial communities on the surfaces of endotracheal tubes used to administer mechanical ventilation to neonates, and the prevalence of *P. aeruginosa* on these endotracheal tubes was as high as 86% (Li et al., 2015b). Furthermore, we hypothesized that the high prevalence of *P. aeruginosa*, combined with the presence of other microorganisms on the same tubes in ventilator-associated pneumonia (VAP) neonates, is attributed to the regulation of the quorum sensing (QS) signaling pathway (Li et al., 2015b).

The pathogenicity of *P. aeruginosa* infection is attributed to the various virulence factors expressed by this bacterium as well as its ability to form biofilms, which are controlled by QS (Lee and Zhang, 2015). QS is a form of bacterial cell-to-cell communication, and QS refers to the ability of bacteria to respond to small signaling molecules that are secreted by various microbial species (Saipriya et al., 2020). These signaling molecules are referred to as autoinducers (AIs). When the concentration of an AI accumulates to a particular threshold, the QS system is activated *via* extracellular receptors. Autoinducer-2 (AI-2), which is considered the universal bacterial signal, can regulate both intra- and interspecies communication (Wang et al., 2019). AI-2 is produced in a manner that is dependent on the LuxS genes, and it is the primary QS molecule produced by many gram-positive and gram-negative bacteria. However, *P. aeruginosa* lacks the LuxS genes; thus, it cannot produce AI-2, although it does have the ability to detect and respond to AI-2 (Duan et al., 2003). Previously, we and other researchers found that AI-2, as well as the AI-2-producing bacterium *Streptococcus mitis*, can affect the behaviors of *P. aeruginosa in vitro* and *in vivo* (Li et al., 2015a; Wang et al., 2016; Li H. et al., 2017). Furthermore, AI-2 can increase mortality rates and exacerbate lung infection in a mouse model of acute *P. aeruginosa* pneumonia (Li H. et al., 2017), but the immunological mechanism underlying this pathogenesis is not fully understood.

In addition to their impact on bacterial biofilm formation and virulence, QS molecules also exert a regulatory effect on the host immune response (Fteita et al., 2018). Interleukin-17A (IL-17A), which is a primary inflammatory cytokine, is produced by many cell types, such as Th17 cells, γδT cells, and innate lymphoid cells, and mediates the recruitment of inflammatory cells, such as neutrophils (Chung et al., 2021). Th17 cells, which are the main producers of IL-17A, differentiate from common naïve CD4+ T cells. STAT3 is a key downstream signal transduction molecule that is involved in Th17-cell differentiation. A review of the recent literature showed that IL-17A can act as a double-edged sword during lung infections (Gurczynski and Moore, 2018). On the one hand, IL-17A protects the host against lung infections. Kudva et al. (2011) found that IL-17A-deficient mice were more susceptible to developing bacterial pneumonia caused by *Staphylococcus aureus* and *Staphylococcus pneumoniae* than wild-type (WT) mice. However, IL-17A can impair host tolerance during chronic airway infection with *P. aeruginosa* (Bielen et al., 2017). Clinical data have shown that the IL-17A levels in the lavage fluids of *P. aeruginosa*-infected patients are significantly higher than those in the lavage fluids of non-infected patients (Decraene et al., 2010; Lorè et al., 2016). However, the effects of AI-2 on the IL-17A production induced by acute *P. aeruginosa* infection are not fully understood.

Herein, we aimed to investigate the effects of AI-2 on *P. aeruginosa* infection *in vivo* using a mouse model of pulmonary infection (both WT mice and IL-17A-deficient mice), and to explore the roles of the AI-2-IL-17A pathway in *P. aeruginosa* infection. This work may help to identify a new strategy to combat *P. aeruginosa* lung infections.

## Materials and methods

### Mice

All the experiments were conducted in compliance with relevant guidelines and regulations, and the animal study was approved by the Animal Care and Use Committee of Chongqing Medical University. Male WT C57BL/6 mice aged 6–8 weeks were purchased from Chongqing Medical University (Chongqing, China). Male IL-17A^−/−^ mice (C57BL/6 background, 6–8 weeks old) were a kind gift from Professor Yibing Yin (Chongqing Medical University, Chongqing, China). All the mice were maintained in specific pathogen-free cages and were given free access to sterile food and water at the Laboratory Animal Center of Chongqing Medical University.

### Bacterial strains and culture conditions

*Pseudomonas aeruginosa* PAO1 (ATCC 27853) and a *P. aeruginosa* strain harboring mutants in the QS genes *lasR* and *rhlR* (*P. aeruginosa* Δ*lasR rhlR*) were grown and maintained on Luria–Bertani (LB) plates or in LB broth at 37°C with agitation (200 rpm). After washing with phosphate-buffered saline (PBS), the bacterial concentration was adjusted to an optical density of 0.25 at 600 nm (OD600). The bacteria were then diluted with PBS or AI-2 and directly used in experiments. The chemically synthesized AI-2 was obtained from Omm Scientific company (Dallas, TX, USA). The concentration of AI-2 used in this study was 10 nM, and this concentration was chosen according to our previous results (Li H. et al., 2017).

### Mouse model of acute *P. aeruginosa* lung infection

The acute lung infection model was established following the method described in our previous publications (Li H. et al., 2017). Briefly, mice were infected by intratracheal instillation of 0.05 mL of bacterial suspension (1 × 10^7^colony-forming units/mouse) under anesthesia with pentobarbital sodium. PBS or 10 nM AI-2 was administered to the control group in the same manner. These mice were sacrificed by cervical dislocation 24 h after infection, and the lungs were harvested for further analysis.

### Hematoxylin and Eosin (H & E) staining

WT mice were anesthetized with pentobarbital sodium 24 h after *P. aeruginosa* PAO1 infection, and then, the lungs were harvested. For histopathological analysis, lung tissues were embedded in 10% neutral buffered formalin for 48 h, and the 5-μm paraffin-fixed sections were cut and stained with hematoxylin-eosin (H&E; Sigma). This process was repeated along the entire lung. Light microscopy (Nikon Eclipse 55i, Japan) was used to identify inflammatory cells. Lung pathological scores were calculated based on the degree of alveolar inflammation; the parameters used for this calculation included alveolar congestion, alveolar cell exudation, alveolar wall thickening, and edema, as previously described (Li J. et al., 2017).

### Determination of IL-17A concentrations and Th17 cell population in WT mice

Twenty-four hours after *P. aeruginosa* PAO1 infection, WT mice were anesthetized with pentobarbital sodium, and the lungs were harvested and homogenized using a homogenizer (Changzheng Co., Chongqing, China) at 4°C. Lung tissue lysates were centrifuged, and 100 μL supernatant per well was further analyzed by enzyme-linked immunosorbent assay (ELISA). The concentrations of the cytokine IL-17A in the lungs were determined by using mouse cytokine ELISA kits (Sizhengbai, Beijing, China) according to the manufacturer's instructions.

For staining Th17 cells, lung specimens were minced, digested and then filtered to generate single-cell suspensions. Lung cells were stimulated at 37°C for 5.5 h with Cell Activation Cocktail containing Brefeldin A (BioLegend) and then labeled with a FITC-conjugated anti-mouse CD4 antibody. Before the cells were stained with a PE-conjugated anti-mouse IL-17 antibody (BioLegend), they were permeabilized with intracellular staining fixation/permeabilization buffer (BioLegend). The stained cells were analyzed using an LSRFortessa cell analyzer (BD, New York, USA). The data were analyzed with FlowJo software (TreeStar), and the percentage of Th17 cells relative to the total CD4+ T cell population was statistically quantified.

### Immunoblotting analysis

Proteins were extracted from lung tissues using RIPA buffer [Cell Signaling Technology (CST), USA], and the concentrations were quantified using a BCA protein assay reagent (Beyotime, Nanjing, China) according to the manufacturer's instructions. Equal amounts of protein extracts were resolved by 8 or 15% sodium dodecyl sulfate–polyacrylamide gel electrophoresis (SDS–PAGE) and then transferred to polyvinylidene difluoride (PVDF) membranes (Millipore, Billerica, MA). The membranes were subsequently incubated with primary antibodies, including rabbit anti-mouse STAT3 polyclonal antibodies (1:1,000, CST, USA), rabbit anti-mouse p-STAT3 polyclonal antibodies (1:1,000, CST, USA), and rabbit anti-mouse GAPDH polyclonal antibodies (1:5,000, Proteintech, China) at 4°C overnight. The membranes were washed and incubated with horseradish peroxidase (HRP)-conjugated goat anti-rabbit secondary antibodies (1:5,000, goat, zsgb-bio, China) at room temperature for 1 h. The protein bands were visualized using the enhanced chemiluminescence method (GE Healthcare Life Sciences, Little chalfont, UK) and quantified by using Quantity One software (Bio-Rad, Hercules, CA). GAPDH was used as the internal reference.

### Cytokine array analysis of IL-17A*^−/−^* mice with lung infections

Given the positive results obtained in the WT mice, IL-17A^−/−^ mice were used to identify the role of IL-17A in *P. aeruginosa* infection in loss-of-function experiments. The mouse model of lung infection was consistent with that established with WT mice, as described above. After the mice were anesthetized with pentobarbital sodium 24 h after *P. aeruginosa* PAO1 infection, the lungs were harvested. For cytokine array analysis, lung tissues were homogenized, and the secreted cytokines were measured with a RayBiotech mouse cytokine array c-1000 according to the manufacturer's instructions.

### Analysis of cytokine concentrations in WT mice infected with *P. aeruginosa* Δ*lasR rhlR*

To determine the mechanism by which AI-2 participates in IL-17A production, a *P. aeruginosa* Δ*lasR rhlR* strain was used to infect WT mice. The mice were anesthetized with pentobarbital sodium 24 h after *P. aeruginosa* Δ*lasR rhlR* infection. The concentrations of the cytokines transforming growth factor-β(TGF-β), IL-6 and IL-17A in the lungs were determined using mouse cytokine ELISA kits (Sizhengbai, Beijing, China) according to the manufacturer's instructions.

### Statistical analysis

GraphPad Prism 8.0 (GraphPad Software, CA, USA) was used to conduct all the statistical analyses and generate the figures. The data were expressed as the mean and standard deviation. Analysis of variance (ANOVA) was used to evaluate the significance of differences between all groups, and Tukey's honest significant difference (HSD) test was used for pairwise comparison. *P* < 0.05 was considered to be statistically significant.

## Results

### AI-2 exacerbated *P. aeruginosa*-induced lung inflammation

**Figure 1:**
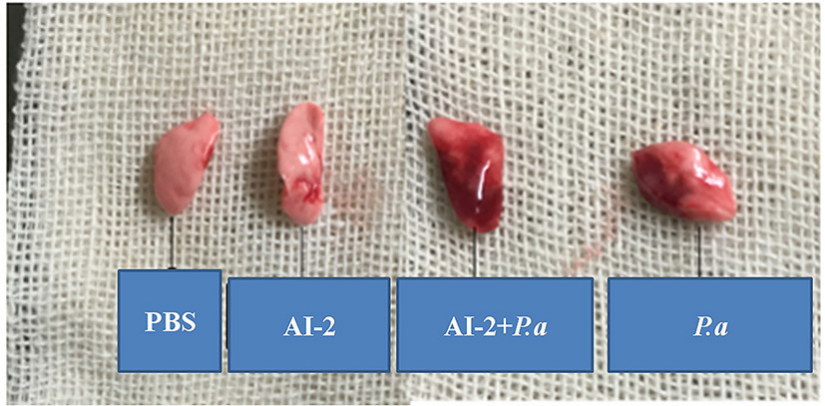
Gross photo of mice's lungs after infection. PBS, phosphate-buffered saline group; AI-2, Autoinducer-2 group; AI-2+ *P.a*, AI-2+ *P. aeruginosa* PAO1 group; *P.a, P. aeruginosa* PAO1 group.

**Figure 2:**
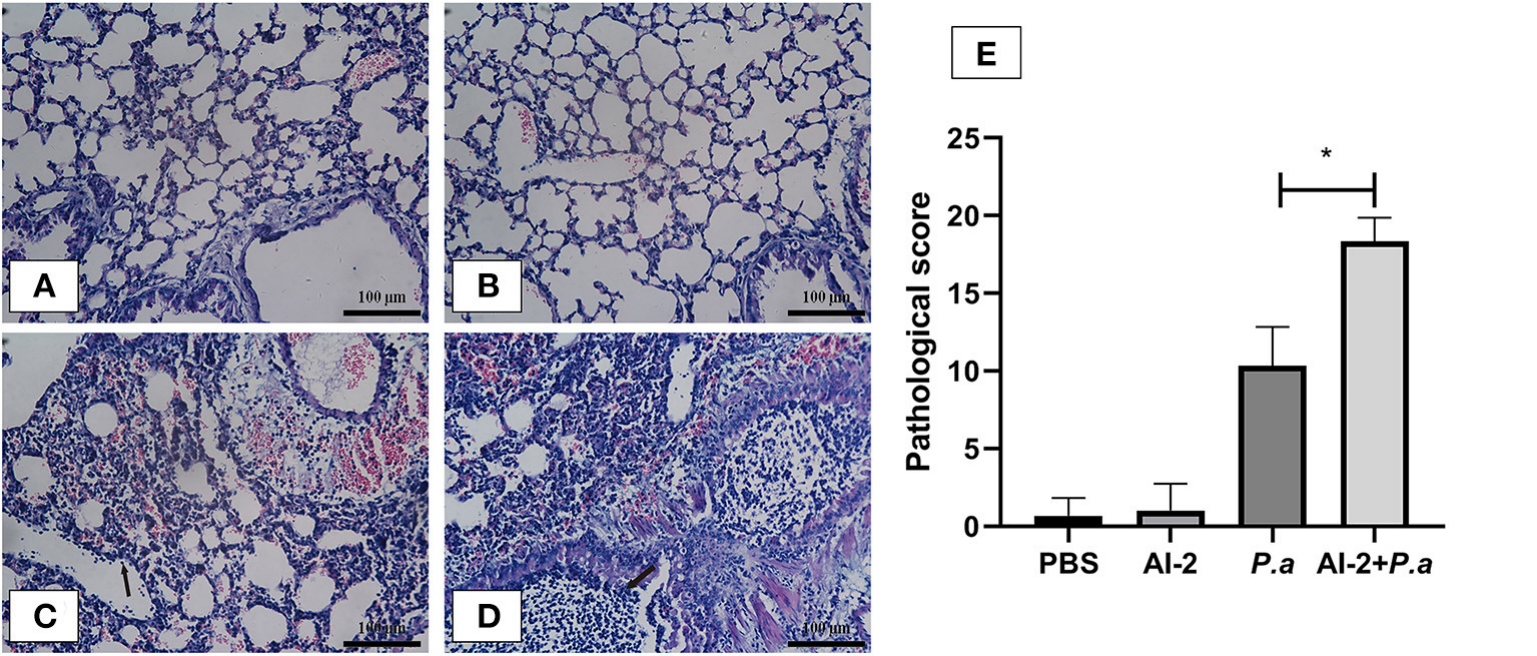
Lung histological examination after infection. Sections of lungs stained with hematoxylin-eosin at 24 h post infection were shown (magnifications, ×400). Black arrows indicate the leukocytes. Scale bar = 100 μm. **(A)** PBS group; **(B)** AI-2 group; **(C)** *P. aeruginosa* PAO1 group; **(D)** *P. aeruginosa* PAO1+AI-2 group. Lung pathological score **(E)** was also analyzed. One of at least three independent experiments with three mice in each group was shown. Results are shown as mean ± SD. *P < 0.05.

To evaluate the effects of AI-2 after *P. aeruginosa* infection, mice were infected by intratracheal instillation of 0.05 mL of bacterial suspension, and the mouse lung tissues were harvested after 24 h. After PAO1 infection, the lung tissues of the mice increased in volume and exhibited a dark red surface color, patchy bleeding and pus spots (Figure 1). Compared to the *P. aeruginosa* PAO1 group (*P.a*), the AI-2+*P.a* group exhibited a greater bacterial burden and more serious pathological changes. However, the lung tissues in the PBS group and AI-2 group were smooth, ruddy and soft, with good elasticity and no obvious hyperemia. As shown in Figure 2, H&E-stained slides from the AI-2+*P.a* group showed that large numbers of inflammatory cells, especially leukocytes, accumulated in the lung tissues. The alveolar spaces were filled with tissue fluid, and the lung structures were damaged. In addition, the inflammatory score of the AI-2+*P.a* group was significantly higher than that of the *P.a* group (Figure 2E). These results demonstrated that AI-2 could exacerbate *P. aeruginosa* lung inflammation.

### AI-2 augmented the levels of IL-17A and Th17 cells in *P. aeruginosa* PAO1-induced pneumonia

**Figure 3:**
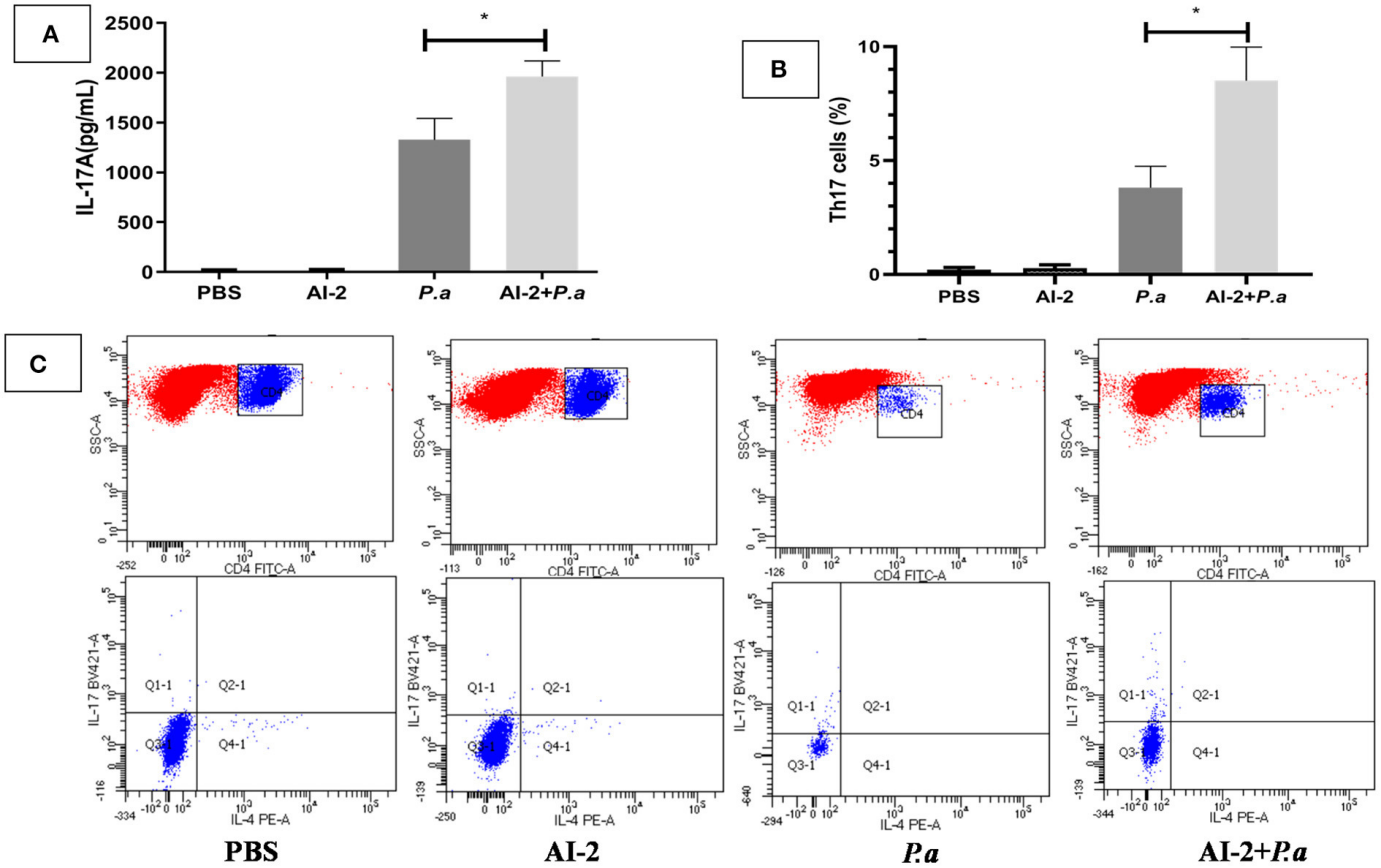
The expression of IL-17A and Th17 cells in *P. aeruginosa* pneumonia. The mice were infected with *P. aeruginosa* PAO1 and AI-2 + *P. aeruginosa* PAO1, respectively. Lungs were collected at 24 h post infection. **(A)** Each sample was analyzed by an IL-17A ELISA kit. **(B)** Statistical analysis of Th17 cells in each group of the mice's lungs. **(C)** Representive flow cytometry analysis graphs of Th17 cells. One of at least three independent experiments with three mice in each group was shown. Results are shown as mean ± SD. *P < 0.05. *P.a, P. aeruginosa* PAO1 group; AI-2+ *P.a*, AI-2+ *P. aeruginosa* PAO1 group.

**Figure 4:**
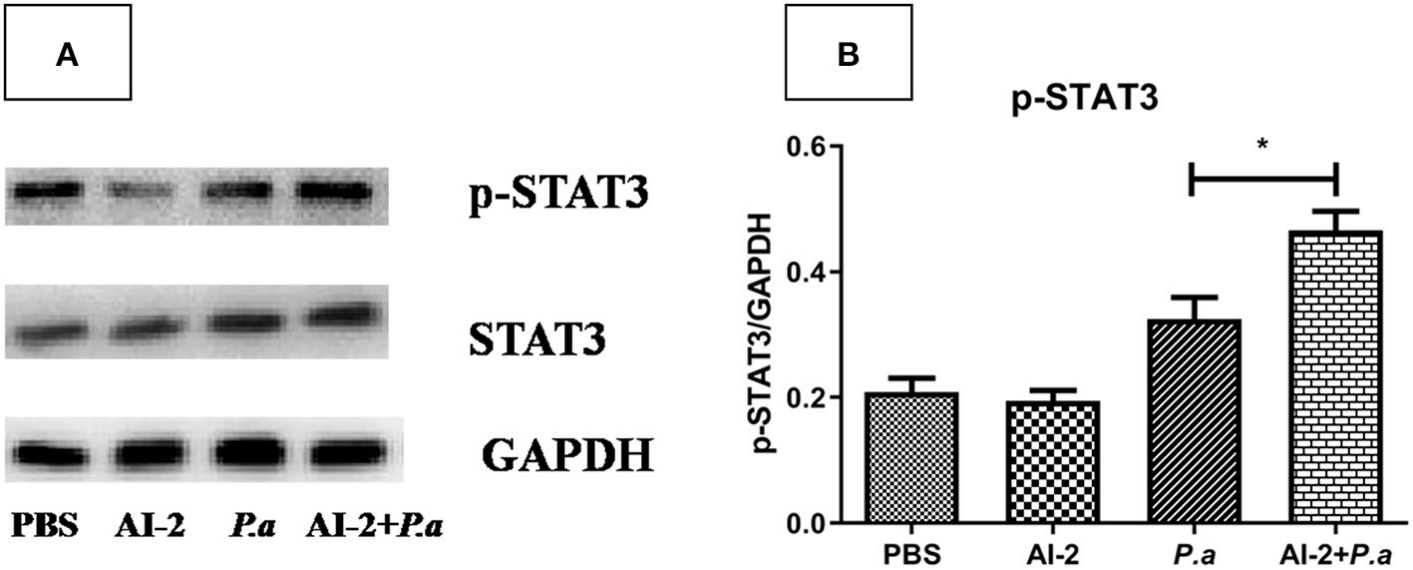
pSTAT3 and STAT3 expression in lungs from mice infected with *P. aeruginosa*. **(A)** Representative immunoblot graphs with pSTAT3, STAT3, and GAPDH expression from mice infected with *P. aeruginosa*. **(B)** Significantly higher intensity ratios of pSTAT3/GAPDH in lungs of AI-2+*P.a* group than those in *P.a* group. One of at least three independent experiments with three mice in each group was shown. Results are shown as mean ± SD. *P < 0.05. PBS, phosphate-buffered saline group; AI-2, Autoinducer-2 group; *P.a, P. aeruginosa* PAO1 group; AI-2+ *P.a*, AI-2+ *P. aeruginosa* PAO1 group.

As IL-17A has been shown to play a devastating role in lung infections, we aimed to examine whether AI-2 can increase the levels of IL-17A and proportion of Th17 cells in the lungs after PAO1 infection. ELISA-based assays and flow cytometry were performed. As shown in Figure 3, the levels of IL-17A in the lungs were significantly higher in the AI-2+*P.a* group than in the *P.a* group (1964.3 ± 145 vs. 1327.4 ± 95 pg/mL, *P* < 0.05; Figure 3A). We further explored the changes in the proportion of Th17 cells, which are the main producers of IL-17A, in the lungs. Compared to the *P.a* group, the AI-2+*P.a* group exhibited higher levels of Th17 cells in terms of both the percentages and absolute numbers in the lungs (*P* < 0.05; Figures 3B,C). Previous studies have demonstrated that STAT3 signaling plays a crucial role in Th17-cell differentiation (Zhou et al., 2018); therefore, we explored the expression of pSTAT3 and STAT3 in lung cells and found that there were significantly higher pSTAT3/GAPDH ratios in the AI-2+*P.a* group than in the *P.a* group (*P* < 0.05; Figure 4). These results demonstrated that AI-2 could increase the levels of IL-17A and the proportions of Th17 cells in the lungs after *P. aeruginosa* PAO1 infection.

### AI-2 exacerbated *P. aeruginosa* lung infection *via* the IL-17A pathway

**Figure 5:**
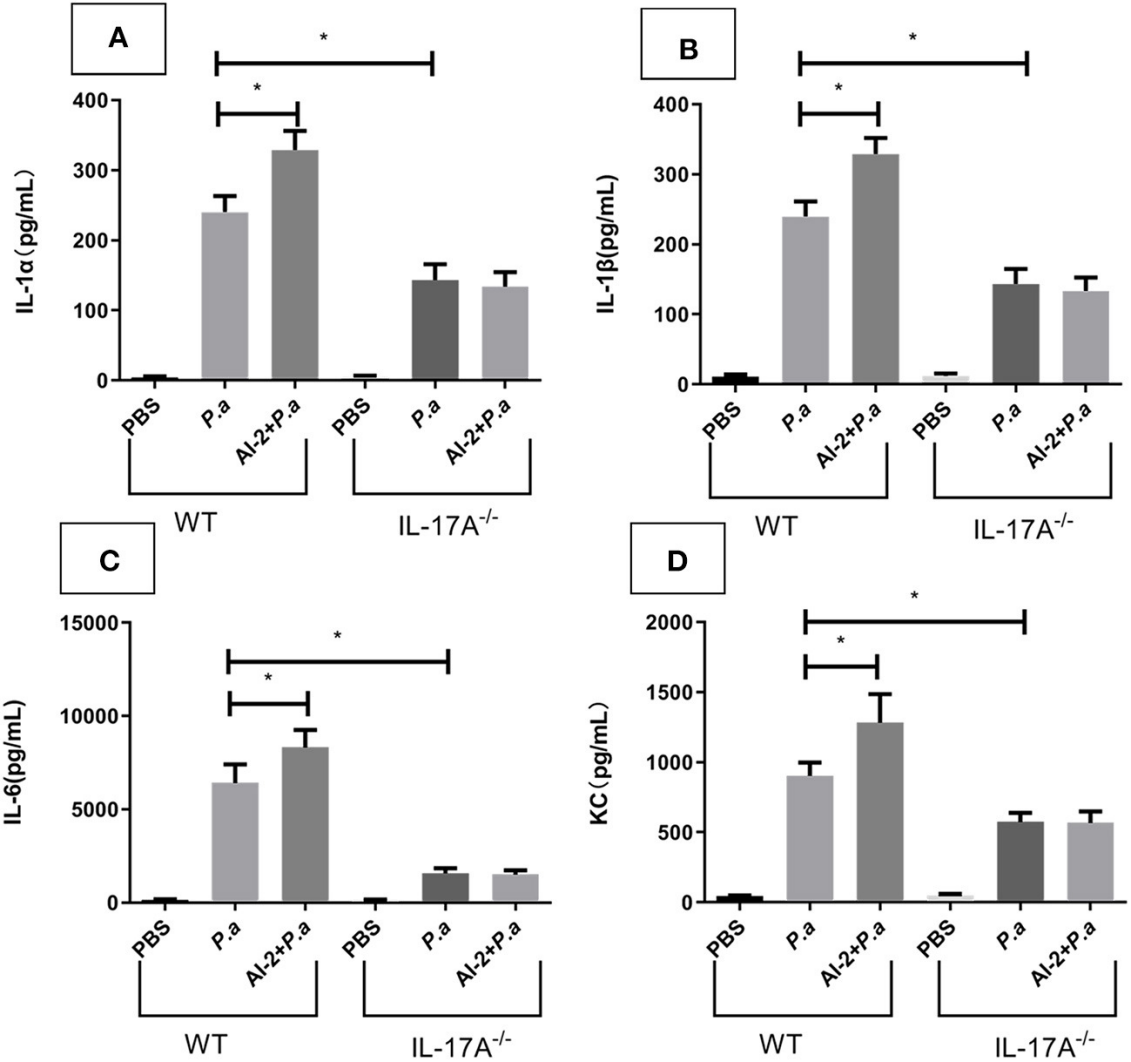
Cytokine array analysis for IL-17A^−^/^−^ mice in lungs from mice infected with *P. aeruginosa* PAO1. WT and IL-17A^−^/^−^ mice were intratracheal instillation with PBS, *P. aeruginosa* PAO1 and AI-2+*P. aeruginosa* PAO1, respectively. Lungs were collected at 24 h post infection. Lung tissues were prepared for cytokine array analysis. IL-1α **(A)**, IL-1β **(B)**, IL-6 **(C)**, and KC **(D)** levels present in the lungs of the respective groups were shown. One of at least three independent experiments with three mice in each group was shown. Results are shown as mean ± SD. *P < 0.05. *P.a, P. aeruginosa* PAO1 group; AI-2+ *P.a*, AI-2+ *P. aeruginosa* PAO1 group.

Having shown that AI-2 could exacerbate the expression of IL-17A, we next investigated the effects of IL-17A activity on *P. aeruginosa* lung infection using IL-17A-deficient mice. WT and IL-17A^−/−^ mice were intratracheally infected with viable *P. aeruginosa* PAO1 or sham-infected with PBS (as a negative control) and analyzed after 24 h. Similar to the ELISA results (Figure 3A), cytokine array analysis showed that pulmonary infection with *P. aeruginosa* PAO1 resulted in significantly increased expression of IL-17A in the WT mice (*P* < 0.05), whereas IL-17A was not detectable in the infected IL-17A^−/−^ mice 24 h after infection (data not shown). There were no significant differences in the amount of IL-17A between the *P.a* group and AI-2+*P.a* group when IL-17A^−/−^ mice were studied. The concentrations of IL-1α, IL-1β, IL-6 and the chemokine KC were significantly reduced in the infected IL-17A^−/−^ mice compared with the infected WT mice (*P* < 0.05; Figure 5). In accordance with the IL-17A data, there were no significant differences in the levels of IL-1α, IL-1β, IL-6, or the chemokine KC between the *P.a* group and AI-2+*P.a* group of IL-17A^−/−^ mice. These results in IL-17A^−/−^ mice indicate the important role of IL-17A in *P. aeruginosa* infection in the context of AI-2.

### AI-2 activated the IL-17A pathway *via* the QS system during *P. aeruginosa* lung infections

**Figure 6:**
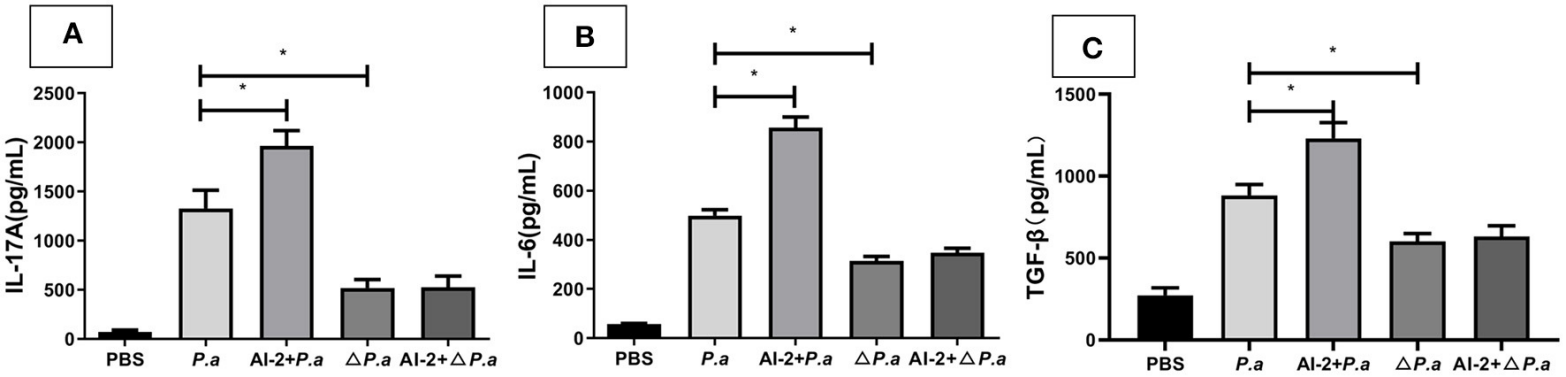
Cytokine concentrations in WT mice infected by *P. aeruginosa* Δ*lasR rhlR* strain. WT mice were intratracheal instillation with PBS, *P. aeruginosa* PAO1, AI-2+*P. aeruginosa* PAO1, *P. aeruginosa* Δ*lasR rhlR* and AI-2+*P. aeruginosa* Δ*lasR rhlR*, respectively. Lungs were collected at 24 h post infection. Lung tissues were prepared for ELISA analysis. IL-17A **(A)**, IL-6 **(B)**, TGF-β **(C)** levels present in the lungs of the respective groups were shown. One of at least three independent experiments with three mice in each group was shown. Results are shown as mean ± SD. *P < 0.05. *P.a, P. aeruginosa* PAO1 group; AI-2+*P.a*, AI-2+*P. aeruginosa* PAO1 group; Δ*P. a, P. aeruginosa* **Δ***lasR rhlR* group; AI-2+Δ*P.a*, AI-2+*P. aeruginosa* **Δ***lasR rhlR* group.

AI-2, which is a QS signal, is thought to substantially contribute to the virulence of *P. aeruginosa*, but the mechanism by which AI-2 impacts the IL-17A pathway remains unknown. Here, we investigated whether AI-2 mediates the production of IL-17A and other cytokines during lung infection with WT *P. aeruginosa* or a *P. aeruginosa* strain harboring mutations in QS genes. To this end, we infected WT mice with a WT *P. aeruginosa* PAO1 (*P.a*) or *P. aeruginosa* Δ*lasR rhlR* strain (Δ*P.a*) and determined the concentrations of IL-17A and other cytokines in the lungs. The level of IL-17A in the lungs was significantly reduced in the Δ*P.a* group compared with the *P.a* group (519.9 ±113 vs. 1327.4 ± 102 pg/mL, *P* < 0.05; Figure 6A). However, there were no statistically significant differences betweenthe Δ*P.a* group and the AI-2+Δ*P.a* group (*P* > 0.05). Similar to the IL-17A results, the concentrations of IL-6 and TGF-β also were not different between the Δ*P.a* group and the AI-2+Δ*P.a* group (*P* > 0.05; Figures 6B,C). These results demonstrated that AI-2 could activate the IL-17A pathway *via* the QS system during *P. aeruginosa* lung infections.

## Discussion

*Pseudomonas aeruginosa* infections, as hospital-acquired infections, have become a great threat, especially in critically ill or immunocompromised patients (Gellatly and Hancock, 2013; Hashimoto et al., 2022). The emergence and frequency of antibiotic resistance in *P. aeruginosa* underlie the urgent need to develop novel antimicrobial agents, such as immunotherapeutic agents. The main findings of this study demonstrated that AI-2 could upregulate the STAT3/IL-17A signaling pathway in Th17 cells in response to *P. aeruginosa* lung infection. We also found that this function of AI-2 might be attributed to the regulation of QS by *P. aeruginosa*. These results suggest that the immune responses to *P. aeruginosa* infection that are mediated by IL-17A are not protective and that an excessive host inflammatory response induced by AI-2 can cause lung damage.

Although IL-17A has been shown to play both protective and pathogenic roles in chronic *P. aeruginosa* infections (Gurczynski and Moore, 2018), its role in acute *P. aeruginosa* infections, such as acute respiratory distress syndrome and VAP, seems to be unclear (Orlov et al., 2017; De Winter et al., 2019; Wong et al., 2021). In our study, we demonstrated that IL-17A was a proinflammatory cytokine that exacerbated pulmonary injury using WT and IL-17A^−/−^ mouse models. IL-17A is an important cytokine that is involved in the recruitment of neutrophils for the clearance of bacteria, but continuous neutrophil recruitment and excessive release of proteases, such as neutrophil elastase (NE) and matrix metalloproteinase-9 (MMP-9), by neutrophils lead to excessive extracellular matrix degradation and lung injury (Beroun et al., 2019). Our data showed that the concentration of KC, which is a chemokine that recruits neutrophils, was significantly elevated in the AI-2+*P.a* group compared with the *P.a* group, which was consistent with the elevated expression of IL-17A observed in this study. Th17 cells, which are the main producers of IL-17A, are a cell subset characterized by a unique transcriptional profile that is dependent on STAT3 transduction pathways (Chen et al., 2019). The current results demonstrated that Th17 cell numbers and STAT3 and p-STAT3 expression were significantly increased in the AI-2+*P.a* group compared with the *P.a* group. During *P. aeruginosa* lung infections, STAT3 activation has been demonstrated to be essential for enhanced Th17 responses and sustained neutrophilic airway inflammation (Halwani et al., 2017). When STAT3 is genetically deleted in CD4+ T cells, neither naturally occurring Th17 cells nor Th17-dependent autoimmunity occurs (Milner et al., 2008).

The immune response in eukaryotic organisms can be activated by QS signaling molecules (Fteita et al., 2018). Additionally, bacteria communicate and respond to cell density by releasing QS molecules (Jeong et al., 2015). QS is known to play critical roles in biofilm formation and virulence factor production. We previously found that AI-2 could increase *P. aeruginosa* PAO1 biofilm formation and virulence factor production by interfering with *P. aeruginosa* QS systems (Li et al., 2015a; Li H. et al., 2017). Little is known about the function of AI-2 in modulating the behaviors of the host. Pyocyanin, which is a virulence factor of *P. aeruginosa*, can increase TGF-β1 secretion (Wang et al., 2021), while elastase, which is another virulence factor of *P. aeruginosa*, can increase IL-6 secretion (Li et al., 2021). Both TGF-β and IL-6 are crucial cytokines in the differentiation of CD4+ T cells into Th17 cells. In our study, the levels of IL-17A, TGF-β, and IL-6 in the lungs were significantly elevated in mice infected with the WT *P. aeruginosa* PAO1 strain but were significantly reduced in mice infected with the mutant *P. aeruginosa lasR rhlR* strain. Therefore, AI-2 may activate the IL-17A pathway *via* the QS system of *P. aeruginosa* during lung infections *via* pyocyanin and elastase secretion. In fact, many studies have also shown the presence of AI-2 in humans, particularly in inflammatory bowel disease patients and major depressive disorder patients (Raut et al., 2013; Fu et al., 2020; Medina-Rodriguez et al., 2020), and suggested that AI-2 directly promotes Th17-cell differentiation and that fecal IL-17A levels are increased in patients with major depressive disorder (Medina-Rodriguez et al., 2020). However, the study on the effects of AI-2 in those experiments was focused on intestinal Th17 cells rather than pulmonary Th17 cells. Further studies are needed to confirm whether such effects exist in our study. Another limitation to our study is that we could perform more auxiliary experiments, such as lung fluorescence microscopy, to label Th17 cell to support our conclusions in the future.

In summary, this study shows that AI-2 significantly increased the levels of IL-17A and Th17 cells *via* QS systems in a mouse model of acute *P. aeruginosa* pneumonia. Our results suggest an important role of IL-17A in *P. aeruginosa* infection in the presence of AI-2, suggesting that IL-17A may be a potential target for adjunctive therapy, which could be administered in combination with antimicrobial therapy, to treat patients with acute *P. aeruginosa* lung infections.

## Data availability statement

The original contributions presented in the study are included in the article/supplementary material, further inquiries can be directed to the corresponding author.

## Ethics statement

The animal study was reviewed and approved by the Animal Care and Use Committee of Chongqing Medical University.

## Author contributions

HL and LT conceived and designed this study. HL, XL, and QA performed the experiments. HL and XL analyzed the data and wrote the paper. LT revised the manuscripts. All authors contributed to the article and approved the submitted version.

## Funding

This study was supported by the Natural Science Foundation of Chongqing (No. cstc2020jcyj-msxmX0253).

## Conflict of interest

The authors declare that the research was conducted in the absence of any commercial or financial relationships that could be construed as a potential conflict of interest.

## Publisher's note

All claims expressed in this article are solely those of the authors and do not necessarily represent those of their affiliated organizations, or those of the publisher, the editors and the reviewers. Any product that may be evaluated in this article, or claim that may be made by its manufacturer, is not guaranteed or endorsed by the publisher.
